# Single-cell and spatial transcriptomics reveal changes in cell heterogeneity during progression of human tendinopathy

**DOI:** 10.1186/s12915-023-01613-2

**Published:** 2023-06-06

**Authors:** Weili Fu, Runze Yang, Jian Li

**Affiliations:** Department of Orthopedics, Orthopedic Research Institute, West China Hospital, Sichuan University, Chengdu, 610041 China

**Keywords:** Tendinopathy, Single-cell RNA-seq, Spatial RNA-seq, Heterotopic ossification

## Abstract

**Background:**

Musculoskeletal tissue degeneration impairs the life quality and motor function of many people, especially seniors and athletes. Tendinopathy is one of the most common diseases associated with musculoskeletal tissue degeneration, representing a major global healthcare burden that affects both athletes and the general population, with the clinical presentation of long-term recurring chronic pain and decreased tolerance to activity. The cellular and molecular mechanisms at the basis of the disease process remain elusive. Here, we use a single-cell and spatial RNA sequencing approach to provide a further understanding of cellular heterogeneity and molecular mechanisms underlying tendinopathy progression.

**Results:**

To explore the changes in tendon homeostasis during the tendinopathy process, we built a cell atlas of healthy and diseased human tendons using single-cell RNA sequencing of approximately 35,000 cells and explored the variations of cell subtypes’ spatial distributions using spatial RNA sequencing. We identified and localized different tenocyte subpopulations in normal and lesioned tendons, found different differentiation trajectories of tendon stem/progenitor cells in normal/diseased tendons, and revealed the spatial location relationship between stromal cells and diseased tenocytes. We deciphered the progression of tendinopathy at a single-cell level, which is characterized by inflammatory infiltration, followed by chondrogenesis and finally endochondral ossification. We found diseased tissue-specific endothelial cell subsets and macrophages as potential therapeutic targets.

**Conclusions:**

This cell atlas provides the molecular foundation for investigating how tendon cell identities, biochemical functions, and interactions contributed to the tendinopathy process. The discoveries revealed the pathogenesis of tendinopathy at single-cell and spatial levels, which is characterized by inflammatory infiltration, followed by chondrogenesis, and finally endochondral ossification. Our results provide new insights into the control of tendinopathy and potential clues to developing novel diagnostic and therapeutic strategies.

**Supplementary Information:**

The online version contains supplementary material available at 10.1186/s12915-023-01613-2.

## Background

Musculoskeletal tissue degeneration impairs the life quality and function of many people, especially seniors and athletes. Nowadays, the concept of national sports has been deeply rooted in people’s hearts. But with the increase in their daily exercise and poor exercise style, more and more people develop tendinopathy. Tendinopathy is one of the most common diseases in sports medicine affecting both athletes and the general population, with the clinical presentation of long-term recurring chronic pain and decreased activity tolerance [1]. Tendinopathy poses a significant clinical challenge especially in the field of orthopedics and sports medicine, accounting for approximately 30% of the general practice musculoskeletal consultations and leading to severe social economic burden [2, 3]. Generally, tendinopathy commonly affected the lower and upper extremities of our body. The incidence of lower limb tendinopathy is reported to be 10.52 per 1000 person-years, while the incidence of affected rotator cuff tendons reaches up to 5.5% [4]. The prevalence of tendinopathy leads to permanent deficits in function and life in both athletic and non-athletic individuals of all ages. The onset of tendinopathy is insidious, and the treatment is challenging. The diagnosis of tendinopathy is mostly based on clinical symptoms, and their accuracy and precision remain ambiguous [5]. Currently, there is no unified and effective treatment. Although conservative treatment and operative techniques can improve symptoms to a certain extent, the treatment is still full of uncertainty [6–8]. The reason of which is that the etiopathogenesis of tendinopathy has not been thoroughly elucidated.

According to the report, inflammatory responses and biomineralization influence the cellular microenvironment in the tendon and gradually result in the occurrence of tendon injury [9, 10]. Nevertheless, the changes in the microenvironment in lesioned tendons remain largely unclear. A comprehensive understanding of the variations that occurred in the diseased tendon is crucial for preventing the occurrence and development of the disease in young and middle-aged patients and for relieving symptoms in elderly patients with tendinopathy. To date, transcriptome changes in injured tendons have been reported in several studies by using bulk RNA sequencing [11–13], but these cannot reveal the changes in the cell types in the tendon and the changes in gene expression of each cell subtype during the disease. It has been reported that the cells within the tendon are heterogeneous, and many cell types, such as vascular endothelial cells and immune cells, can contribute to tendon injury [14].

As a result of continuous technological innovation and constantly reducing costs, single-cell RNA sequencing (scRNA-seq) has been increasingly used as a powerful alternative to bulk RNA sequencing in the past few years. Key advantages of scRNA-seq over bulk methods are the capability to reveal rare and complex cell clusters, discover regulatory relationships between genes, and track the development trajectories of different cell lineages [15, 16]. Spatial RNA sequencing (spRNA-seq) is a recently developed revolutionary technology. It combined the advantages of the comprehensive analysis of bulk RNA sequencing and in situ hybridization to provide complete transcriptome data with spatial information [17]. ScRNA-seq combined with spRNA-seq has been used to decipher the spatial structure of the liver [18–20] and liver diseases such as primary hepatocellular carcinoma [21]. To our knowledge, studies regarding scRNA-seq combined with a spRNA-seq application for exploring the pathogenesis of tendinopathy are very limited. Garcia-Melchor et al. using scRNA-seq revealed the interaction between T cells and tenocytes promotes tendinopathy [22]. Akbar et al. used scRNA-seq combined with spRNA-seq to characterize the disturbance of the immune microenvironment in human tendon disease [23]. However, the existing data cannot completely and clearly decipher the different cell subsets in tendons of human tendinopathy, the influence of stromal cells such as vascular endothelial cells on the course of the disease, and the spatial information between stromal cells and tenocytes in the diseased state.

In this study, we collected normal tendons and lesioned tendons and then performed scRNA-seq and spRNA-seq. We constructed a single-cell transcriptome atlas for all major subtypes of normal and diseased tendons and found the differentiation trajectories of stem cells unique to the diseased group. We further revealed the changes in the microenvironment during tendinopathy and elucidate the cell types and key genes and molecular events contributing to this transformation. These findings will help us more thoroughly understand the progression of tendon injury and provide potential targets for tendinopathy therapies.

## Results

### scRNA-seq and cellular landscape in human normal and diseased tendons

**Fig. 1:**
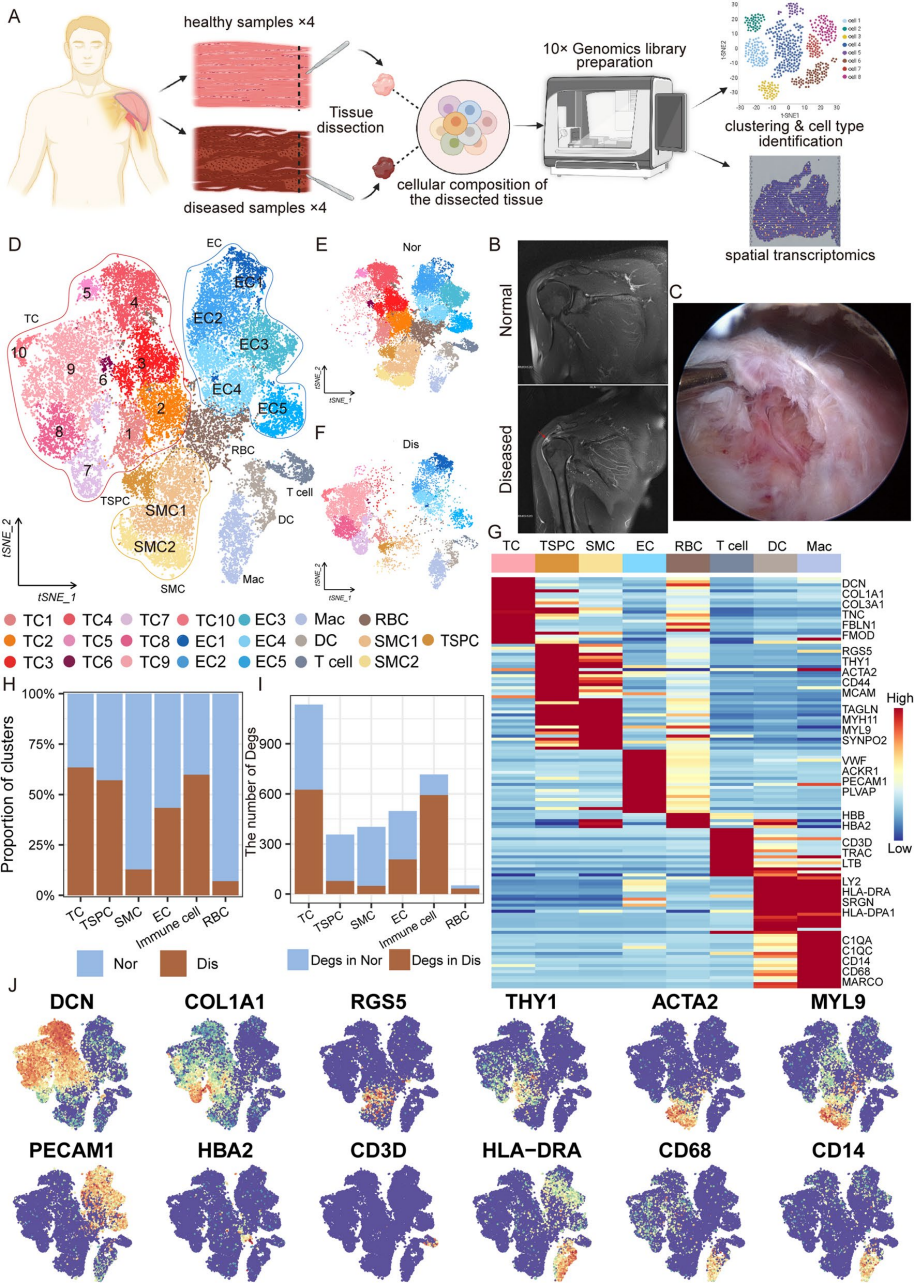
Single-cell RNA-seq reveals major cell classes in the human tendon. **A** The overall workflow of our research program. **B** MRI photographs of typical normal (up) and lesioned tendon (down). **C** A photograph of a typical lesioned tendon under an arthroscope. **D** t-SNE visualization of all cell clusters in collected tendons. **E**, **F** t-SNE visualization of the donor origins in normal/diseased samples. **G** Heatmap of selected marker genes in each cell cluster. **H** The percentages of the identified cell classes in normal/diseased tendons. **I** Number of differentially expressed genes (DEGs) in each cell type of normal/diseased status. **J** Feature plots of expression distribution for selected cluster-specific genes. Brighter colors indicate higher expression levels

To dissect the cellular heterogeneity and explore the molecular profiles of the tendinopathy, we performed scRNA-seq on tendons of rotator cuff injury, a paradigm of tendinopathy, and normal tendon tissues. We randomly collected four healthy tendons and four diseased tendons from the patient cohort (Additional file 1: Table 1). These specimens were next dissociated and prepared for sequencing as shown in the overall workflow (Fig. 1A). The normal supraspinatus tendon showed moderate and homogeneous signal intensity on the T2 phase of magnetic resonance imaging (MRI) (Fig. 1B). When the rotator cuff is injured, the patient showed limited movement of the affected limb, inflammatory edema occurred in their supraspinatus tendons, presenting high signal intensity on the T2 phase of MRI and adhesion and scar hyperplasia under arthroscopy (Fig. 1B, C). After strict quality control steps removing low-quality cells and inferred doublets, we obtained transcriptomes of 34,736 cells (diseased tendon: 11,798; normal tendon: 22,938, Additional file 2: Fig. S1A–C).

Unsupervised t-distributed stochastic neighbor embedding (t-SNE) clustering revealed 8 cell compartments, including 10 tenocyte (TC) subpopulations (TC1, TC2, TC3, TC4, TC5, TC6, TC7, TC8, TC9, TC10), tendon stem/progenitor cell (TSPC), 5 endothelial cell subpopulations (EC1, EC2, EC3, EC4, EC5), T cells, dendritic cell (DC), macrophage (Mac), 2 smooth muscle cell (SMC) subpopulations (SMC1, SMC2), and red blood cell (RBC) (Fig. 1D). From t-SNE plots of different sample sources, we could find that cluster composition, especially in tenocytes, differed markedly between the normal and diseased groups (Fig. 1E, F). These 8 cell lineages were determined based on the established lineage-specific marker genes in Fig. 1G. We identified cell groups as tenocytes if they expressed a high level of tendon-related genes such as *DCN*, *COL1A1*, and *TNC* and cell groups with high expression levels of *VWF*, *PECAM1*, and *PLVAP* as the endothelial cell. Immune cells were recognized using high *PTPRC*; macrophage markers *CD68*, *CD14*, and *MARCO*; antigen presentation markers *HLA-DRA*, *HLA-DPA1*, and *SRGN*; and T cell markers *CD3D* and *LTB*. Cells expressing a high level of muscle-related genes such as *MYL9*, *MYH11*, and *TAGLN* were identified as smooth muscle cells (SMC). In addition, there are a small group of cells separated from other cells in both normal and diseased tendons, which has high expression levels of well-established canonical markers of TSPC such as *ACTA2*, *RGS5*, *THY1*, and *MCAM* (Fig. 1J and Additional file 3: Table S2).

We next analyzed the proportions of these cell compartments in normal and diseased tendons. The cell lineages of normal and diseased groups showed distinct relative cell number ratios (Fig. 1H). Increased proportions were observed for endothelial cells, smooth muscle cells, and red blood cells in normal tissue. The proportions of tenocytes, TSPC, and immune cells (including T cell, DC, and Mac) were slightly increased in diseased tissues compared with normal tendons, perhaps resulting from the regeneration and repair of damaged tendons. After that, we explored the number of differentially expressed genes between normal and diseased tendon clusters. The results showed that the tenocytes had the largest difference, suggesting that tenocytes undergo significant changes during the disease process (Fig. 1I).

### Characterization of subpopulations of tenocytes and TSPC between different kinds of tissue states

**Fig. 2:**
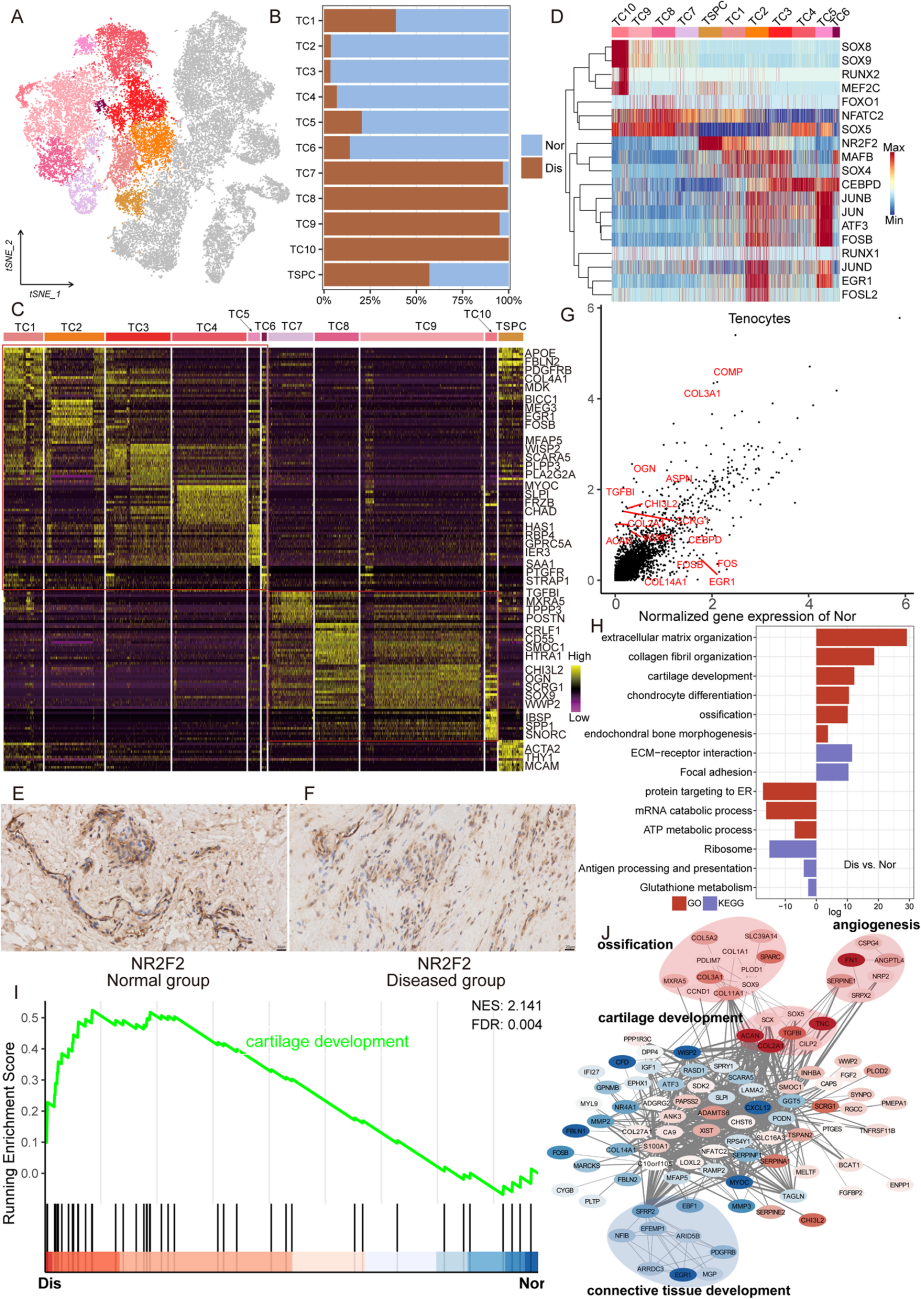
Characterization of tenocyte subclusters and TSPC across different statuses in the human tendon. **A** t-SNE visualization of the subclusters of tenocytes and TSPC. **B** The proportions of 10 tenocyte subpopulations and TSPC in normal and diseased tendons. **C** A cell-level heatmap reveals the normalized expression of DEGs for each cluster defined. **D** Heatmap of gene expression regulation by transcription factors applying SCENIC for each tenocyte subcluster and TSPC. **E**, **F** Immunohistochemical staining of NR2F2 in normal and diseased samples. **G** Volcano plot showing the DEGs between two statues of tenocytes and TSPC. The *x*-axis represents highly expressed genes in normal cells, and the *y*-axis represents highly expressed genes in diseased cells. **H** GO and KEGG enrichment analysis of DEGs in tenocytes and TSPC between normal and lesioned tendons. **I** GSEA enrichment plots for representative signaling pathways upregulated in tenocytes and TSPC of diseased samples, compared to normal samples. **J** Gene–gene interaction networks between DEGs in tenocytes and TSPC of the normal group and tenocytes and TSPC of the diseased group

Because tenocytes, the main cell cluster in the tendon, undergo significant changes in the course of tendon injury, and tenocytes and TSPC are important for tendinopathy pathogenesis, we next performed further analysis of these two cell clusters in all normal and diseased samples (Fig. 2A). As is shown in Fig. 2B, the cell proportions of the tenocyte subpopulations and TSPC in normal tendons differed from diseased tendons. The results illustrated that the proportion of tenocyte subclusters varied greatly between the diseased group and the normal group, while the proportion of TSPC in the normal group was slightly less than in the diseased group. The ratio of TC1-6 in the normal tendon was obviously higher than that in the diseased tendon; however, TC7-10 were essentially tenocyte subsets specific to the lesioned group.

To decipher the characteristics of tenocyte subclusters and TSPC, we analyzed and compared the differentially expressed genes (DEGs) in these 11 clusters (Fig. 2C and Additional file 4: Table S3). From the results, we can conclude that TC1 is a group of resident fibroblasts that function normally as they highly expressed the genes related to tendon growth and differentiation such as *MDK* and *PDGFRB*, and major tendon extracellular matrix (ECM) components such as *FBLN2*, *COL1A1*, and *COL3A1*. TC2 is a cluster of fibroblasts that expressed a high level of *MEG3*, *EGR1*, *DCN*, *COL1A2*, and *FBLN1* responsible for the regulation of tenocyte proliferation and the synthesis of important ECM components. TC3 expressing anti-inflammatory and protective genes such as *PLA2G2A*, *SCARA5*, *PLPP3*, and *GPNMB* is a population of homeostasis-associated fibroblasts with defensive functions. We empirically defined TC4 and TC5 as two clusters of attachment cells at the tendon-to-bone junction [24]. Intriguingly, TC5 is closer to the bone, expressing cartilage lubrication proteins HAS1 and PRG4 and closely related to endochondral ossification, while TC4 is closer to the tendon, expressing fibroblast markers *MYOC* and *IGFBP6* and articular cartilage markers *THBS4*, *CILP*, and *CHAD*. TC6 is a group of inflammation-related fibroblasts with high expression levels of pro-inflammatory genes such as *SAA1*, *PTGFR*, *STEAP1*, and *RARRES1*. We depicted TC7 as a population of pathological repair-related fibroblasts because they highly expressed genes associated with scar healing such as *TPPP3*, *COL3A1*, *COL5A1*, and *DPT*. For the remaining subclusters of tenocytes, we empirically defined TC8 (expressing *CD55*, *CRLF1*, and *SMOC1*) as pre-hypertrophic chondrocytes, TC9 (expressing *OGN*, *SOX9*, and *SCRG1*) as hypertrophic chondrocytes, and TC10 (expressing *IBSP* and *SPP1*) as osteocytes [25]. As for TSPC, they expressed classic stem cell hallmarks such as *ACTA2*, *THY1*, and *MCAM*.

After that, we applied single-cell regulatory network inference and clustering (SCENIC) to evaluate the differences in expression levels of transcription factors (TFs) in TSPC and tenocytes (Fig. 2D). From the results, we found that *NR2F2* was highly activated in TSPC, while its expression was low or absent in other tenocyte clusters. This is consistent with reports that *NR2F2* can maintain the stem cell phenotypes, while inactivation of *NR2F2* in stem cells can promote their differentiation into osteoblasts [26, 27]. The results of immunohistochemistry further demonstrated the expression of TSPC-specific TF *NR2F2* in the normal and diseased groups, suggesting the existence of TSPC in these two groups (Fig. 2E, F). We can also see that *SOX5*, a chondrogenic associated TF, was highly expressed in TC7–10 and TC4–5, and *NFATC2* that can also promote chondrogenic differentiation [28] was highly activated in tenocyte clusters specific to the diseased group. In addition, osteogenic-related TFs *SOX9* and *RUNX2* were highly activated in TC10, which further demonstrated that TC10 was a cluster of bone cells. In normal tenocyte clusters (TC1–6), TFs *MAFB*, *SOX4*, *CEBPD*, and *JUN* family motifs were activated, and we inferred that these TFs are crucial for normal tendon growth and development.

### Disease-associated cell subpopulation changes

For further in-depth analysis of changes in tenocytes during the disease process, we compared the overall differences of tenocytes and TSPC between the normal and degenerated tendons using DEG analysis (Fig. 2G). We identified genes upregulated in the diseased group such as *TGFBI*, *COL3A1*, *OGN*, *COMP*, *SCRG1*, and *ASPN*, which were associated with scar healing, chondrogenesis, and ossification, and genes upregulated in the normal group such as *CEBPD*, *COL14A1*, *FOS*, and *EGR1*, which were consistent with the results of TF analysis in TC1–6 and were related to normal tendon growth and differentiation. Gene Ontology (GO) and Kyoto Encyclopedia of Genes and Genomes (KEGG) analysis were used for the functional interpretation of these DEGs (Fig. 2H and Additional file 5: Table S4). The results illustrated that the increased expression of genes in the lesioned group was enriched for the GO terms “chondrocyte differentiation,” “ossification,” and “endochondral bone morphogenesis” and the KEGG terms “ECM-receptor interaction” and “focal adhesion.” Gene set enrichment analysis (GSEA) also suggested that cartilage development, extracellular matrix organization, and collagen fibril organization were enriched in the diseased tendon (Fig. 2I and Additional file 6: Fig. S2A and B).

After filtering out genes expressed in fewer than 25% of all cells, we investigated the genetic interactions between 1049 genes in normal tenocytes (TC1–6) and diseased tenocytes (TC7–10) (Fig. 2J). We found that genes upregulated in normal tissues such as *EGR1*, *EBF1*, and *SFRP2* are associated with connective tissue development. In contrast, the genes upregulated in diseased tissues such as *SOX9*, *SPARC*, *ACAN*, *COL2A1*, and *FN1* are involved in ossification, cartilage development, and angiogenesis, which suggests that these activities are crucial during the process of rotator cuff injury. Notably, the results of gene co-expression analysis were consistent with the results of enrichment analysis, and according to the report, angiogenesis can promote cartilage development and further facilitate the process of endochondral ossification [29]. These results demonstrated that disease not only changed the proportion of tenocyte subsets but also altered the identities of these subclusters.

### The trajectory of tenocyte transformation in the process of tendon injury

**Fig. 3:**
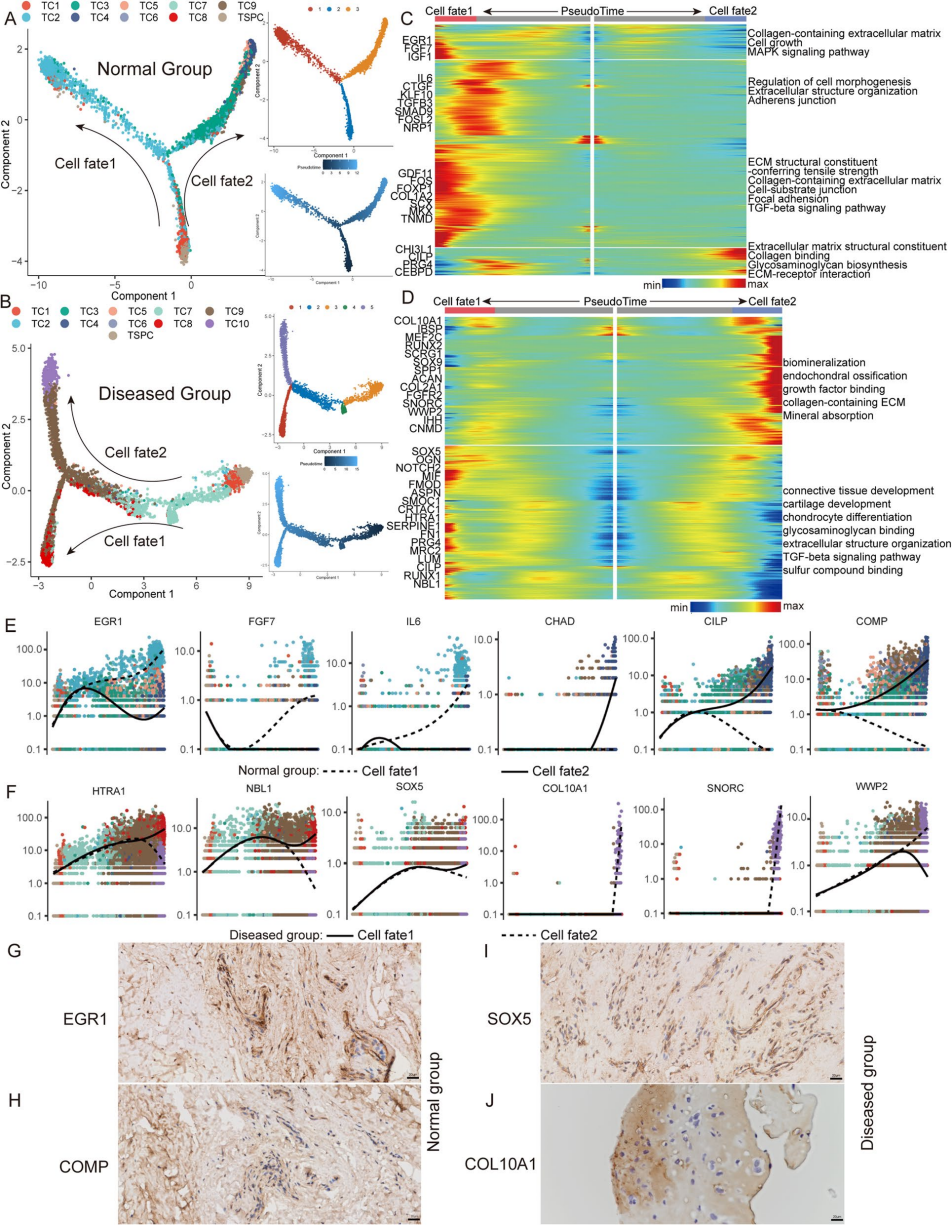
Evolution trajectory and transcriptional fluctuation during tendinopathy progression. **A** Cell trajectory analysis of TSPC differentiation in the normal tendon. **B** Cell trajectory analysis of TSPC differentiation in the diseased tendon. **C** Heatmap showing the expression changes of the highly variable genes along the two cell fates of the trajectory in the normal group. Significantly enriched functional annotations are shown on the right side of the heatmap. **D** Heatmap showing the expression changes of the highly variable genes along the two cell fates of the trajectory in the diseased group. **E**, **F** Representative gene expression levels of different marker genes along TSPC differentiation trajectories of normal and diseased statuses. **G**, **H** Immunohistochemical staining of EGR1 and COMP in the normal tendon. **I**, **J** Immunohistochemical staining of SOX5 and COL10A1 in a lesioned tendon

To explore the critical molecular events governing the cell fate transition during progression from normal to diseased cells, we respectively reconstructed the developmental trajectories of tenocytes in the diseased and healthy groups. We carried out a pseudotime analysis based on Monocle2 and detected non-random expression patterns. The results showed that the diseased and normal groups had two different sets of developmental trajectories with common origination TSPC (Fig. 3A, B). In the normal group, the pseudotime trajectory began with TSPC and then split into two main branches with TC2 and TC4–5 placed at the opposite divergent ends. TC1 and TC3 existed along the trajectory (Additional file 6: Fig. S2D). This means that during normal tendon growth and development, TSPC has two differentiation pathways, one transforming into tenocytes and the other transforming into attachment cells. With this in mind, we tried to explain the molecular dynamics that distinguished the two cell fates (Fig. 3C). The expression profile of cell fate1 showed high expression of tenocyte differentiation genes (*EGR1*, *CTGF*, *IL6*, *SCX*, *MKX*) and different activation of several ECM-associated pathways. Along with the cell fate2 trajectory, we identified high expression of cartilage development-related genes (*CHI3L1*, *CILP*, *PRG4*).

In the diseased group, we constructed a new trajectory containing two termini corresponding to two distinct cell fates. TSPC was found at the origination of the trajectory and then divided into two main branches with TC10 (cell fate2 branch) and TC8–9 (cell fate1 branch) placed at the opposite divergent ends as terminally differentiated cell types. TC7 existed along the trajectory. Of note, TC9 was distributed on both branches, which means that only a fraction of cells in TC9 have the ability to go on to convert to TC10 (Additional file 6: Fig. S2E). From the results, we can infer that in the process of tendinopathy, TSPC cannot differentiate into normal tenocytes but first transform into pathological fibroblasts and then into chondrocytes and osteocytes. The analysis of gene expression dynamics revealed that along with the cell fate1 branch, genes activated at the end of the transition were predominantly involved in the GO terms “cartilage development,” “chondrocyte differentiation,” and “TGF-beta signaling pathway” (*SOX5*, *NOTCH2*, *PRG4*, *SMOC1*). The cell fate2 branch expressed higher levels of genes enriched for the GO terms “biomineralization,” “endochondral ossification,” and “mineral absorption” (*COL10A1*, *IBSP*, *RUNX2*, *SOX9*, *SNORC*, *IHH*) (Fig. 3D).

We selected some typical genes associated with the differentiation of TSPC in normal and lesioned groups and then performed immunohistochemical staining (Fig. 3E–J). The results showed that EGR1 and COMP, which can be found along the differentiation trajectory of normal tissue, are highly expressed in normal tendons, and SOX5 and COL10A1, which can be found along the differentiation trajectory of diseased tissue, are highly expressed in lesioned tendons.

### Blood vessel-derived cells are involved in the process of tendinopathy

**Fig. 4:**
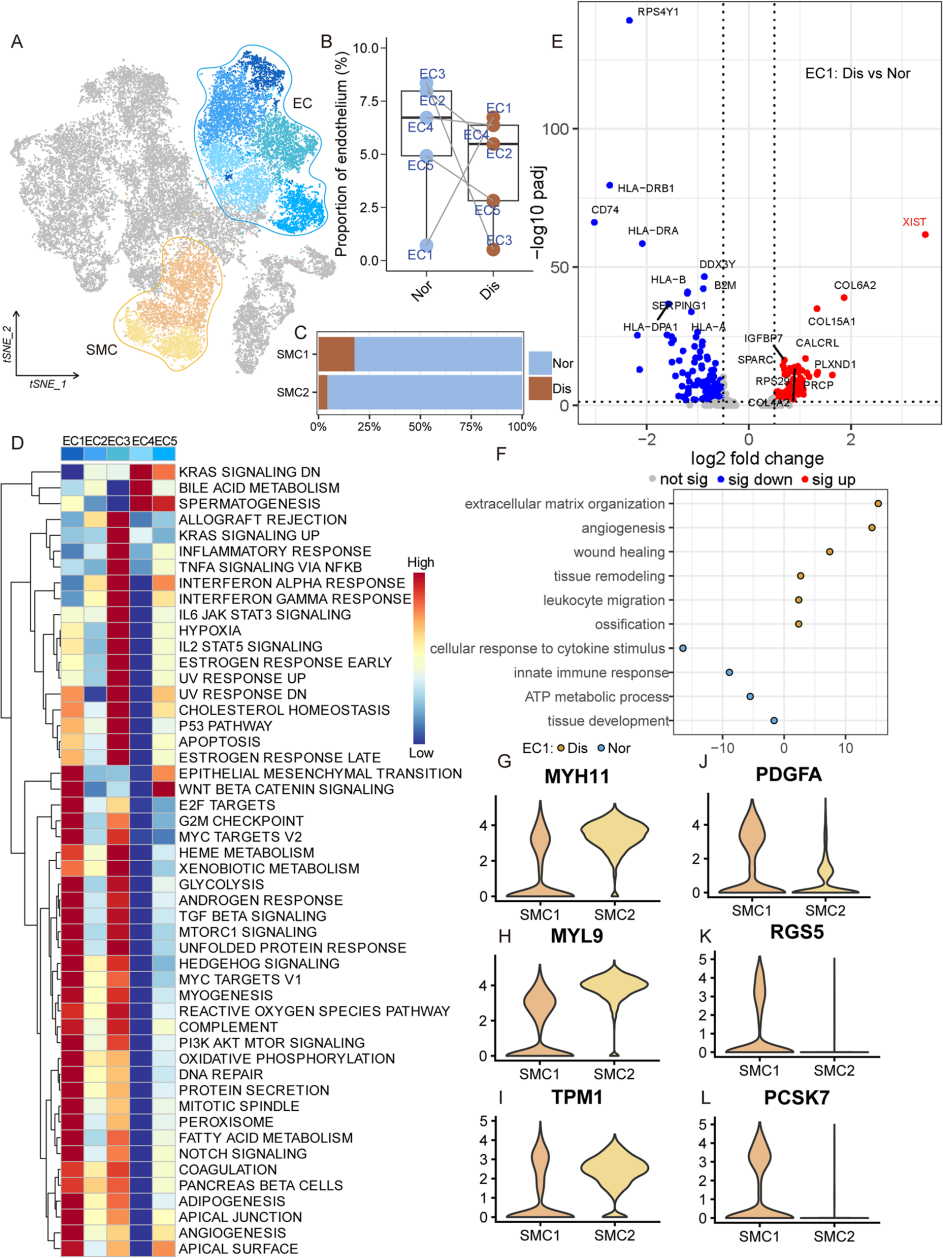
Identification of endothelial cell and smooth muscle cell subclusters in the human tendon. **A** t-SNE visualization of the subclusters of endothelial cells and smooth muscle cells. **B** Summarized subpopulations of endothelial cell percentage changes. **C** The proportion of each subcluster of smooth muscle cells in the lesioned and normal tendons. **D** Heatmap showing the results of enrichment analysis of 50 hallmarker gene sets in EC1-5. **E** Volcano plots displaying the DEGs in EC1 between the normal group and the diseased group. Each dot represented one gene. Red dots, differentially upregulated genes; blue dots, differentially downregulated genes; gray dots, non-differentially expressed genes. **F** GO enrichment analysis of DEGs between normal EC1 and diseased EC1. **G**–**L** Violin plots showing representative marker genes associated with different types of SMC expressed in SMC1 and SMC2

Besides tenocytes, blood vessel-derived cells including endothelial cells (ECs) and smooth muscle cells (SMCs) also have indispensable roles in the occurrence and development of the tendon lesion [30, 31] (Fig. 4A).

In our tendon samples, we identified five clusters of ECs: EC1 highly expressed *APLNR*, *CD93*, *COL4A1*, *EMCN*, *RAMP3*, and *ROBO4* and was related to angiogenesis promotion and inflammation; EC2 expressing a high level of *ACKR1* is a population of venous ECs; EC3 and EC4 expressing high levels of antigen-presenting markers *CD74*, *HLA-A*, and *HLA-E* and immunoregulatory genes *RGS16* and *ICAM1* are immune-related EC populations; and EC5 expressing high levels of *SEMA3G*, *EFNB2*, and *CLDN5* is a population of arteriosus ECs (Additional file 7: Table 5). We next compared the proportions of the five EC subpopulations between normal and diseased tendons. Interestingly, the results showed that only the proportion of EC1 was increased, and the other four groups were decreased in the diseased group compared with the normal group (Fig. 4B). To gain more biological insights underlying these clusters, we used GSEA to compare the expression profiles among these five EC subpopulations. From the results, we can find that some classic osteogenic signaling pathways were highly active in EC1 such as WNT β-CATENIN signaling, TGFβ signaling, NOTCH signaling, and HEDGEHOG signaling (Fig. 4D).

So, we speculated that EC1 was closely associated with the disease progression, and then we compared the EC1 between the healthy/lesioned group and reported a list of DEGs (Fig. 4E). In the normal status, *RPS4Y1*, *HLA-DRA*, *HLA-DRB1*, and *CD74* were upregulated, while in the lesioned status, *XIST*, *COL6A2*, *COL15A1*, and *IGFBP7* were upregulated. *XIST* was ranked first in the diseased group DEGs, which was reported to have promotion effects on angiogenesis, heterotopic ossification, osteoarthritis, and chondrogenic differentiation [32, 33]. DEG enrichment analysis results further illustrated that angiogenesis, tissue remodeling, leukocyte migration, and ossification were activated in EC1 of the diseased tendons (Fig. 4F). These results indicated that EC1 was closely related to the tendon injury and calcification in the diseased group.

In addition, we identified two populations of SMC: SMC1 and SMC2. We empirically identified SMC1 expressing high levels of *PDGFA*, *RGS5*, and *PCSK7* as proliferative SMC and SMC2 expressing high levels of *MYH11*, *MYL9*, and *TPM1* as contractile SMC (Fig. 4G–L). Figure 4C illustrated that the proportion of SMC in normal tissue was significantly higher than that in lesioned tissue and the ratio of SMC1/SMC2 in the diseased group was higher than that in the normal group. According to the reports, under the stimulation of macrophages and inflammatory factors, contractile SMCs will transform into proliferative ones, which is consistent with our results [34]. Secondly, the aging and degeneration of the whole tendon tissue are accompanied by the aging of the blood vessels, and the SMCs in the aging blood vessels will undergo disorder and apoptosis, resulting in decreased elasticity and stiffness of the blood vessels [35]. This could explain why there were more SMCs in the normal tendon than in the diseased tendon.

### Immune cells are involved in the process of tendinopathy

**Fig. 5:**
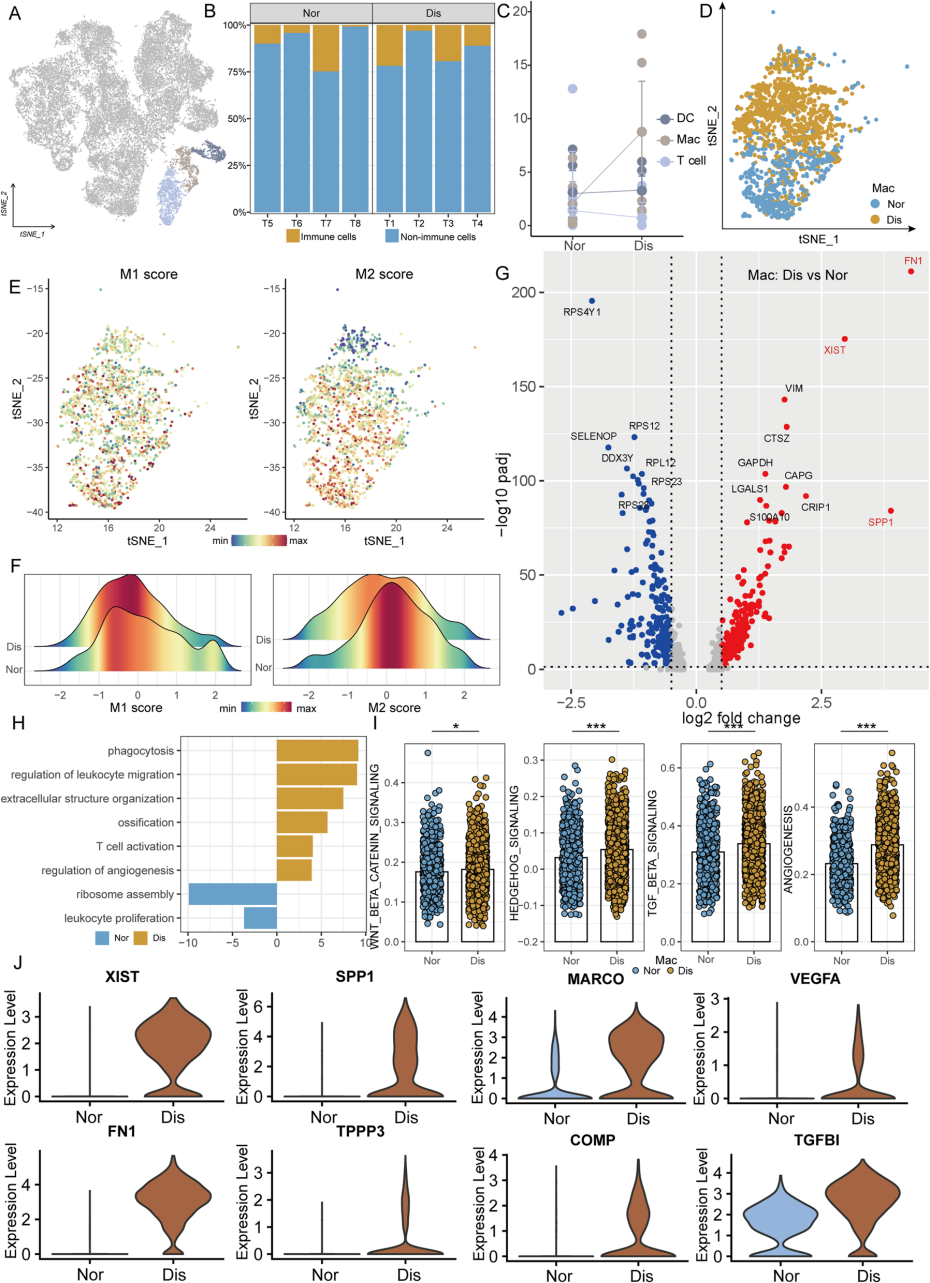
Identification of immune cell subclusters in the human tendon. **A** t-SNE visualization of different types of immune cells in the human tendon. **B** Per-sample bar plots visualize the immune cell percentage changes between the normal and diseased groups. **C** Summarized immune cell percentage changes. **D** t-SNE visualization of macrophages of the normal/diseased group. **E** t-SNE visualization of M1 score (left) and M2 score (right) of macrophages. **F** M1 (left) and M2 (right) score of macrophages in normal/diseased tendon by using gene set variation analysis (GSVA). **G** Volcano plots displaying the DEGs in Mac between the normal group and the diseased group. Red dots, differentially upregulated genes; blue dots, differentially downregulated genes; gray dots, non-differentially expressed genes. **H** GO enrichment analysis of DEGs in Mac between a normal tendon and a diseased tendon. **I** Gene set enrichment analysis (GSEA) of representative signaling pathways related to ossification in Mac of normal and diseased samples. **J** Violin plots showing representative DEGs between macrophages in normal tendons and macrophages in diseased tendons. Two-sided unpaired *t*-test, *p < 0.05, ***p < 0.001

The immune cells are considered as important regulators in tendinopathy and analyzing the immune cells associated with the disease could provide deeper insights into tendinopathy pathogenesis. To investigate immune cell dynamics in the tendon microenvironment, we examined the single-cell transcriptomes of T cells, macrophages (Mac), and dendritic cells (DC) (Fig. 5A). Figure 5B, C illustrated that the proportion of immune cells in the normal group was similar to that in the diseased group, but the number of Mac in the lesioned group increased remarkably compared to the normal group. Therefore, we next focus on analyzing the changes of Mac in the process of disease. From t-SNE plots, we can find that Mac in the normal and diseased groups was distributed in different regions, that is, the cells from the diseased group were mainly concentrated in the upper part of the graph, while the cells from the normal group were mainly concentrated in the lower part of the graph (Fig. 5D). So, we realized that Mac in these two groups had different molecular profiles. M1 and M2 scores of all Mac illustrated that there were more M1-type Mac in the diseased group and more M2-type Mac in the normal group (Fig. 5E, F). It has been reported that M1-type Mac promotes tissue damage, destruction, and inflammation, while M2-type Mac mostly plays a protective role.

DEG analysis was used to further delineate changes in Mac during the disease process, and the results showed that Mac of the diseased group highly expressed *FN1*, *SPP1*, *XIST*, and *VIM* and Mac of the normal group highly expressed ribosomal associated genes *RPS4Y1*, *RPS12*, *RPL12*, and *RPS23* (Fig. 5G). As is shown in Fig. 5H, GO terms “regulation of leukocyte migration,” “extracellular structure organization,” “ossification,” and “regulation of angiogenesis” was enriched in the diseased group. GSEA was used to obtain more biological insights underlying Mac; we can find that osteogenesis-related signaling pathways WNT β-CATENIN signaling, TGFβ signaling, and HEDGEHOG signaling and angiogenesis were significantly upregulated in the diseased tendon (Fig. 5I and Additional file 6: Fig. S2C). Violin plots also proved that genes closely associated with osteogenesis (*SPP1*, *MARCO*, *TGFBI*), chondrogenesis (*FN1*, *XIST*, *COMP*), scar healing (*TPPP3*), and angiogenesis (*VEGFA*) were upregulated in the lesioned tissue (Fig. 5J).

### Cell–cell cross-talk in the tendon microenvironment during tendinopathy progression

**Fig. 6:**
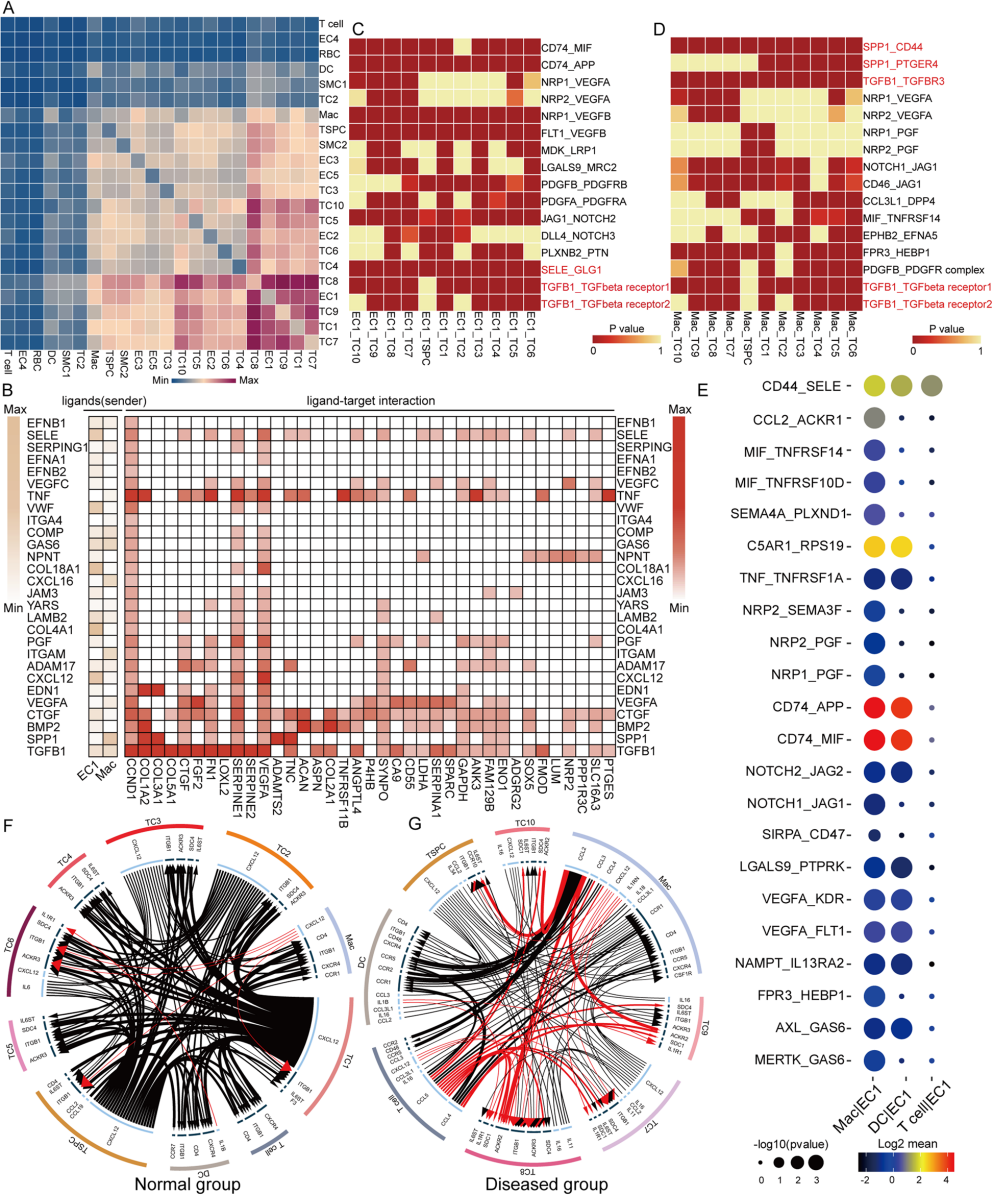
Cell–cell crosstalk during tendinopathy progression. **A** Heatmap depicting the significant interactions among the 22 major cell clusters identified in Fig. 1D. **B** Heatmap showing the potential ligands send from EC1/Mac and their downstream potential target genes expressed in tenocytes and TSPC. **C** Heatmap displaying the potential ligand-receptor pairs related to endochondral ossification identified between EC1 and tenocytes and TSPC. **D** Heatmap showing the potential ligand-receptor pairs associated with endochondral ossification identified between Mac and tenocytes and TSPC. **E** The bubble plot generated by CellPhoneDB showing potential ligand-receptor pairs associated with angiogenesis between immune cells and EC1. **F**, **G** The circo plot showing the potential cytokines and chemokines expressed in immune cells of normal/diseased tendon

Elucidating the explicit interaction among tenocytes, EC, and immune cells in the tendon microenvironment will shed light on the mechanisms of tendon homeostasis and the pathogenesis of tendinopathy. We used CellphoneDB and NicheNet to explore the expression of potential crosstalk signaling molecules based on ligand-receptor interactions. The heatmap depicted that in addition to interactions within tenocyte subsets, the EC1 population in endothelial cells and the Mac population in immune cells showed the most interactions with other cell types, such as tenocytes, suggesting the important role of EC1 and Mac in tendinopathy development (Fig. 6A). Therefore, we used EC1 and Mac as the senders, and then NicheNet was used to detect the expression of ligands of the senders and target genes of the downstream cells (Fig. 6B). Intriguingly, we found that both EC1 and Mac highly expressed *TGFB1* and downstream target genes were more associated with tissue remodeling (*COL3A1*, *CTGF*, *FMOD*, *SERPINE1*), cartilage development (*FN1*, *COL2A1*, *SOX5*), angiogenesis (*VEGFA*), and ossification (*COL5A1*, *ASPN*, *SPARC*). Moreover, *SPP1* expressed by Mac and *BMP2* expressed by EC1 can also act on similar target genes to promote disease progression. The results of CellphoneDB analysis also showed that there are some ligand-receptor pairs associated with ossification and chondrogenesis between EC1 and tenocyte clusters and between Mac and tenocyte clusters and the pairs highlighted in red were the overlapping parts of the results obtained by both CellphoneDB and NicheNet (Fig. 6C, D).

As is shown in Fig. 6E, some ligand-receptor pairs related to angiogenesis such as CD74_APP, CD74_MIF, and CD44_SELE were upregulated between immune cells, especially the Mac, and EC1, suggesting Mac could act on EC1 and promote angiogenesis. This could further explain the increased number of EC1 in diseased tissues compared to normal tissues. Finally, representative ligand-receptor circle figures also indicated that cytokine and chemokine signaling interactions, such as *CCL2*, *CCL3*, *CCL4*, *CCL5*, and *CXCL12*, were significantly increased in the lesioned group compared to the normal group (Fig. 6F, G). It can be inferred that the level of inflammation in the damaged tissues is remarkably higher than that in the normal samples, thus facilitating the process of tendon injury. Taken together, these results indicated that Mac and EC1 altered the microenvironment in the tendon, and they acted on tenocytes to promote the transition of normal tenocytes to chondrocytes and osteocytes, thereby promoting the development of tendinopathy (Fig. 9).

### spRNA-seq of tendon deciphering the molecular variations in the process of tendinopathy

**Fig. 7:**
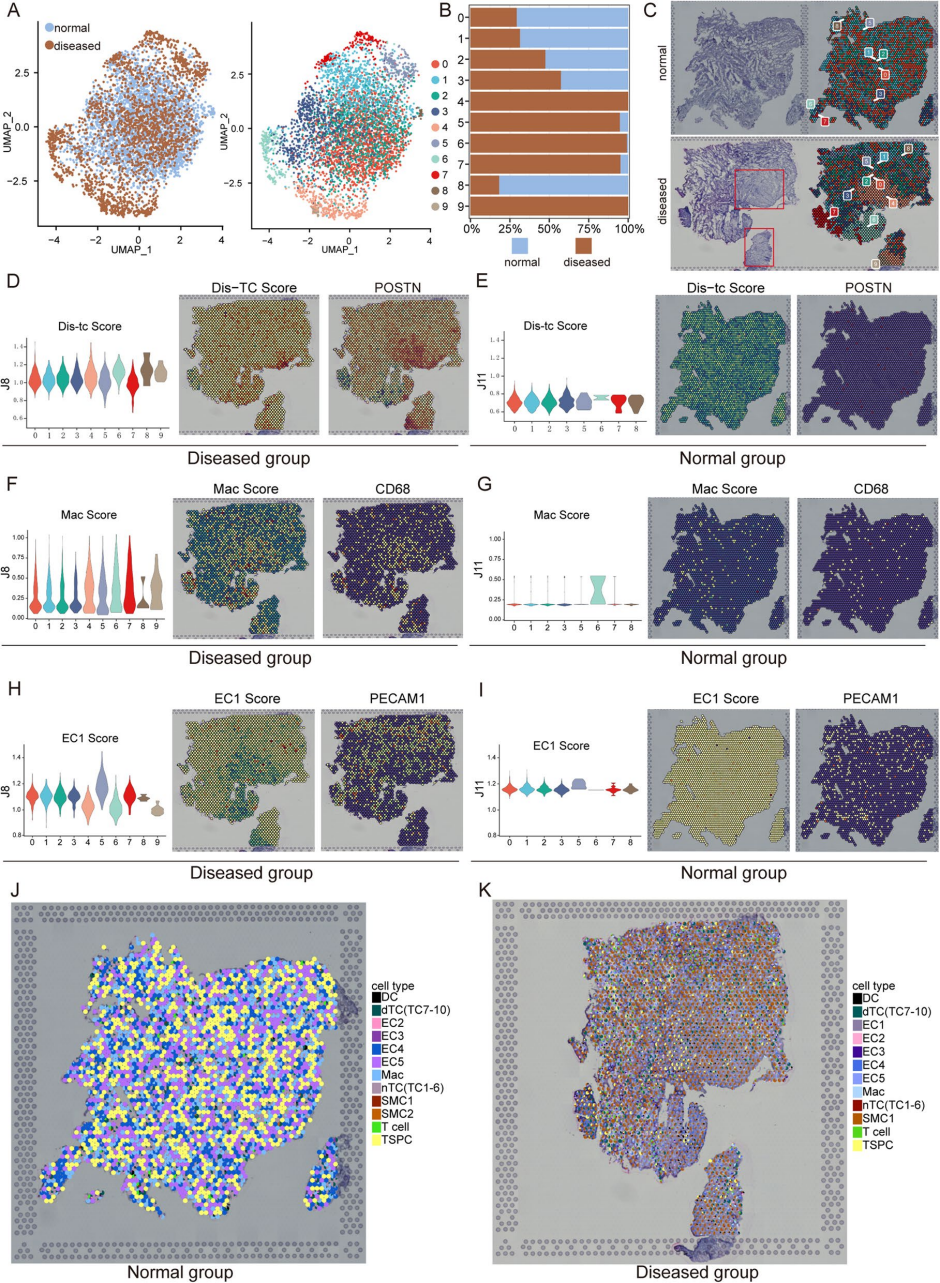
Spatial transcriptome sequencing deciphers the molecular variations during tendinopathy progression. **A** UMAP visualization of the distribution of cells at different sample statuses (left). UMAP visualization of 10 cell clusters in spatial transcriptome sample (right). **B** The proportion of each subcluster in the normal and diseased groups. **C** H&E staining of normal and diseased tendons (left) and distribution of 10 clusters in the tissue space (right). **D** Dis-TC score of 10 clusters in the diseased group (left). Spatial heatmaps showing the Dis-TC score (middle) and the expression level of POSTN (right) in the diseased group. **E** Dis-TC score of 10 clusters in the normal group. Spatial heatmaps showing the Dis-TC score (middle) and the expression level of POSTN (right) in the normal group. **F** Mac score of 10 clusters in the diseased group. Spatial heatmaps depicting the Dis-TC score (middle) and the expression level of CD68 (right) in the diseased group. **G** Mac score of each cluster of the normal group. Spatial heatmaps depicting the Dis-TC score (middle) and the expression level of CD68 (right) in the normal group. **H** EC1 score of each cluster in the diseased group (left). Spatial heatmaps depicting the EC1 score (middle) and the expression level of CD31 (right) in the diseased group. **I** EC1 score of each cluster in the normal group (left). Spatial heatmaps depicting the EC1 score (middle) and the expression level of CD31 (right) in the normal group. **J**, **K** Spotlight plots showing the spatial distribution of different cell populations in normal (**J**) and degenerative (**K**) tendons

For an in-depth, multi-angle interpretation of the molecular changes that occurred in tendinopathy, we performed spRNA-seq on normal and lesioned tendons. By analyzing the results of spRNA-seq, we identified 9 clusters of cells in two types of specimens, and the uniform manifold approximation and projection (UMAP) plots also showed the approximate distribution of these 10 clusters in two samples (Fig. 7A). Combined with the results of subpopulation proportion analysis, we can find that the proportions of clusters 4, 5, 6, 7, and 9 in the diseased group were significantly higher than that in the normal group, suggesting these cell clusters are unique to the diseased specimen (Fig. 7B). The results of HE staining illustrated that the structure of the lesioned tendon was disordered, and the fiber cord-like structure disappeared in some parts. For example, on the right side of the diseased sample, we cannot see the typical fibrous structure, especially the area in the red boxes (Fig. 7C). The distribution of the clusters was also presented in the tissue’s physical space. As shown in Fig. 7C, we found that the clusters in normal and diseased groups had the characteristics of regional distribution. Clusters 4, 5, 6, 7, and 9 can be hardly found in the normal group, while these cell clusters can be easily detected in the diseased group and are concentrated in areas with significant structural disorganization.

**Fig. 8:**
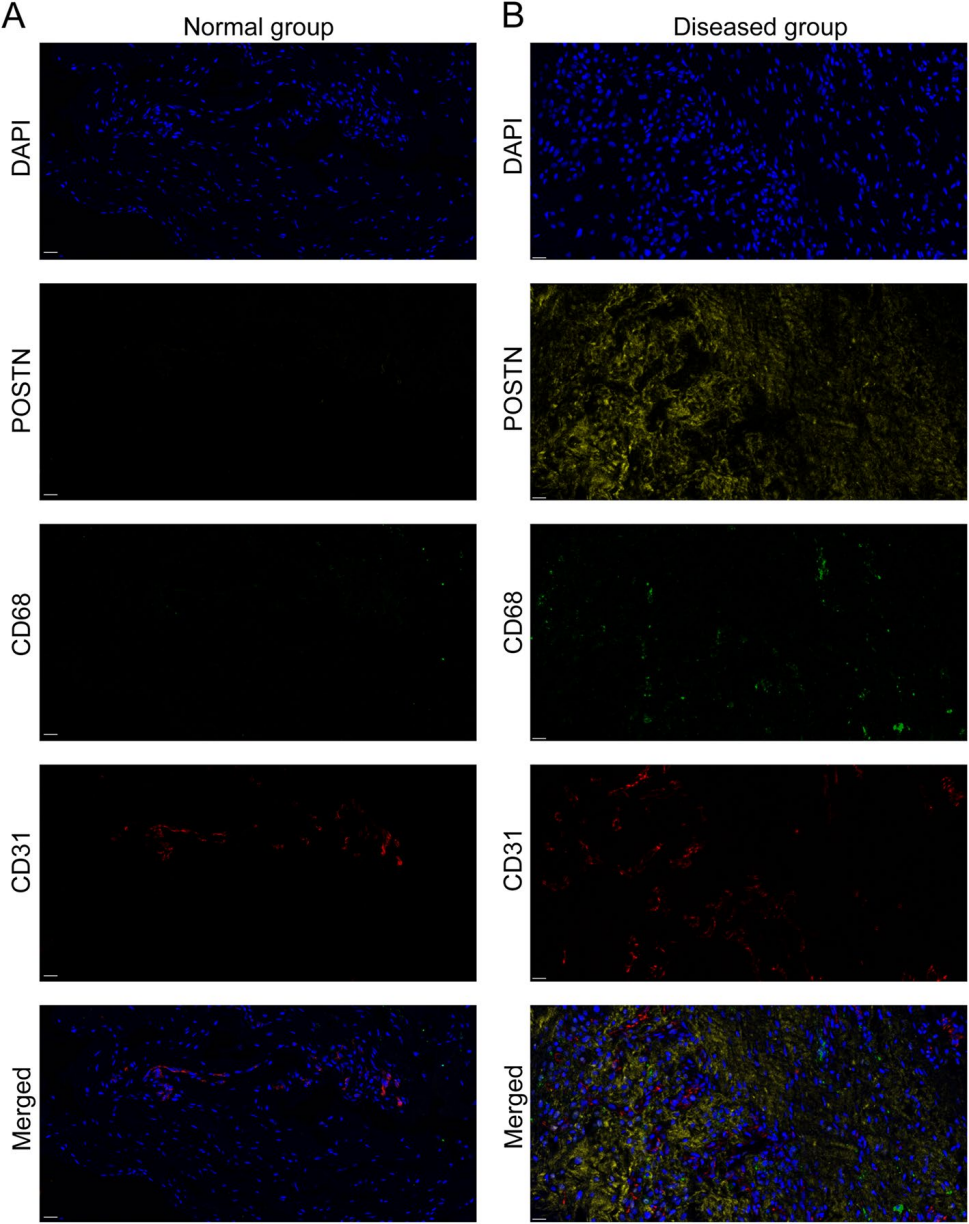
Immunofluorescent staining of human tendons demonstrating the distribution of diseased tenocytes, macrophages, and endothelial cells. **A**, **B** Immunofluorescent staining of POSTN, CD68, and CD31 in normal (**A**) and diseased (**B**) groups (scale bar: 20 µm)

We next used the results of our scRNA-seq analysis to select the gene sets of diseased TC, Mac, and EC1 and then respectively scored different items for the spatial transcriptome sequencing results of normal and diseased tissues (Fig. 7D–I). The results showed that the Dis-TC score, Mac score, and EC1 score in clusters of the diseased group were higher than that of the normal group, suggesting lesioned tissue had much more diseased TC, Mac, and EC1 compared to the normal tissue. Notably, we have got some important spatial information from the results. We discovered that three items, especially Dis-TC and Mac, high-scoring areas were concentrated in similar locations where the tendon has an obvious structural disorder. This implied that the areas with a structural disorder in lesioned tendon have the distribution of diseased TC, Mac, and EC1, and these three types of cells are close together in space. It has been reported that the adjacent location in space is more conducive to the occurrence of mutual communication between cells. Next, in order to better validate the findings obtained from the scRNA-seq, the spotlight was employed to deconvolute the spatially indexed datasets. From the results, we can see the spatial distribution of cell subsets identified by scRNA-seq on normal and degenerative tendon specimens (Fig. 7J, K). Compared with healthy tendon tissue, the cell types at each spatial site were more complex in degenerative tendon tissue. Disease-associated tenocytes (TC7-10) and EC1 were more frequently found in the degenerated sample than in the normal tissue. Meanwhile, dTC (TC7–10), macrophages, and EC1 were often spatially distributed in the same site in degenerated tendons and more likely to have cell interactions, which implied that these cells are closely related to the pathogenesis of the disease. By analyzing the interaction in spatial transcription, we further found that the interaction between cells in the disease samples was stronger than the normal group, which is consistent with the findings of scRNA-seq (Additional file 6: Fig. S2F). According to the results of immunofluorescence and immunohistochemical staining, we can further find that the expression level of POSTN, CD68, and CD31 was upregulated in the diseased tissue (Fig. 8, Additional file 6: Fig. S2G and Additional file 8: Fig. S3A and B). Consequently, the spatial location relationship further demonstrated our conclusion that Mac and EC1 contribute to the development and progression of tendinopathy.

## Discussion

Recent advances in single-cell studies of the human tendon have revealed the dysregulated immune homeostasis during tendinopathy progression [23] and gave a rough picture of the cell types in the tendon [14]. Nevertheless, the accumulated data were insufficient to reveal pathogenic participants in the microenvironment and the key mechanisms leading to the development of the disease. Here, we displayed a high-resolution scRNA-seq dataset of cells in tendons collected from two different tissue statuses to recapitulate crucial transcriptional events in the process of tendinopathy.

Tenocytes are increasingly recognized as central mediators of tendinopathy, and here, we identified 10 tenocyte subpopulations in human normal and diseased tendons by using scRNA-seq. Further cluster analysis suggested that TC7–10 were the diseased group-specific tenocyte clusters. TC7, the main fibroblast in the diseased tissue, highly expressed scar healing-related genes *TPPP3*, *COL3A1*, and *COL5A1* and can perform a pathological repair of the injured tendon. Tendons have a uniaxially arranged collagenous ECM, and this characteristic ECM structure is critical for facilitating force transmission and directly affects the overall mechanical function of the tissue [36]. Type I collagen, the predominant collagen in the tendon, accounts for about 90% of the total collagen content [37]. However, scar healing is dominated by the formation of type III collagen, which changes the structure of the ECM and influences the mechanical strength of the tendon [38]. Interestingly, we found that TC8–9 were clusters of chondrocytes and TC10 was a cluster of bone cells. The results of pseudotime analysis illustrated that the differentiation trajectories of TSPCs in the diseased group were completely different from that in the normal group, and during the course of tendinopathy, transcriptional modules associated with ossification and chondrogenesis were activated, resulting in the transition of TSPC into chondrocytes and osteocytes. The process from pre-hypertrophic chondrocytes (TC8) to hypertrophic chondrocytes (TC9) and finally to osteocytes (TC10) is also consistent with the process of endochondral ossification [39]. Therefore, we realized that the pathological process of tendinopathy is essentially the process of heterotopic ossification (HO) in the tendon. Tendon HO is characterized by endochondral bone formation inside the tendons and is the major histopathological feature associated with advanced tendon injury [40, 41]. Overall, in this study, we have successfully deciphered the important role of tendon HO, a key event in the progression of tendinopathy, and the crucial transcriptional changes governing the cell fate transition during tendon HO at the single-cell level.

**Fig. 9:**
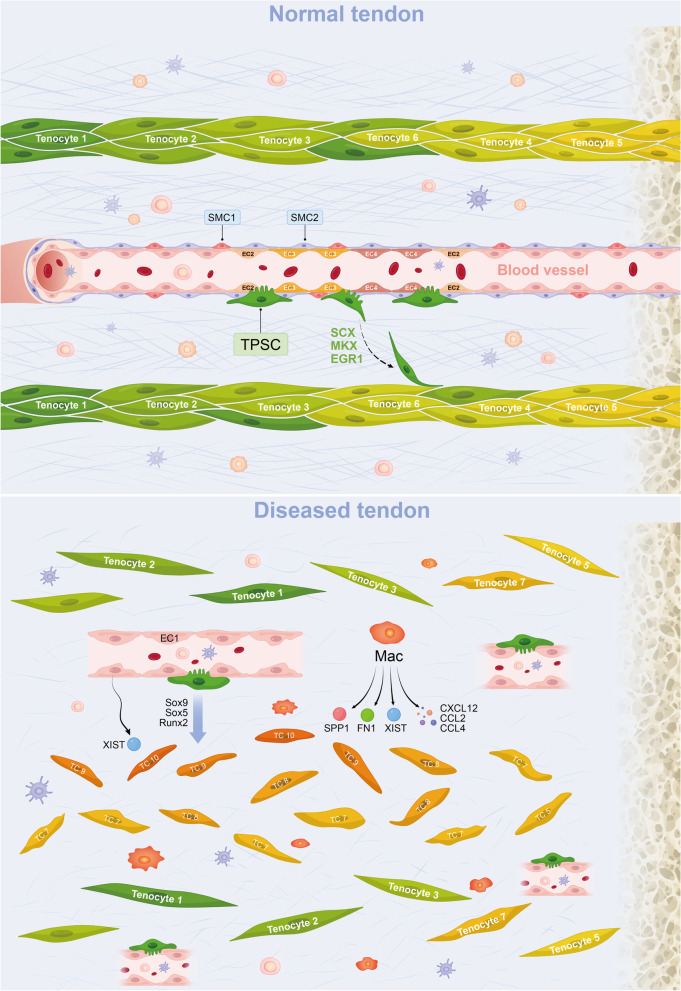
A schematic diagram of the microenvironment changes between the normal and the degenerated meniscus. The upper side visualizes the healthy tendon, where TC1, TC2 and TC3 are dominant tenocyte populations. In this situation, TSPC could differentiate into normal subsets of tenocytes and the aberrant proliferation of blood vessels is inhibited. The lower side visualizes the degenerated tendon where the orchestrated microenvironment balance is broken. In this situation, TSPC could differentiate into chondrocytes and osteocytes, which resulted in HO. The EC1 grew and formed new blood vessels in the diseased area. The vascular permeability increased and allowed more immune cells to infiltrate the degenerated tissue. Proliferating EC1 and Mac can also act on the diseased tenocytes by expressing osteogenic and chondrogenic genes (SPP1, FN1, XIST) to promote disease progression

The microenvironmental homeostasis in the tendon is crucial in controlling the occurrence and development of tendon injury [42, 43]. Tendon’s microenvironment changes significantly from homeostasis when the disease progresses (Fig. 9). In lesioned tendon, we identified two clusters of cells, Mac and EC1, which were closely related to the disruption of microenvironmental homeostasis. EC1 and Mac secreted a variety of cytokines and chemokines that promote the occurrence of chronic inflammation in injured tendons and cause an imbalance in microenvironmental homeostasis. Notably, genes that can promote chondrogenesis and osteogenesis such as *XIST*, *FN1*, *SPP1*, *COL5A1*, and *TGFB1* were upregulated, and ossification-related pathways such as WNT β-CATENIN signaling, TGFβ signaling, and HEDGEHOG signaling were activated in these cells. The results of CellPhoneDB and NicheNet analysis demonstrated that EC1 and Mac can interact with tenocytes to promote endochondral ossification in tendon through ligand-receptor pairs such as SPP1_PTGER4, TGFB1_TGFβ receptor1, and BMP2_CCND1. The results of spRNA-seq further confirmed that EC1, Mac, and the diseased tenocytes were concentrated in the structurally disordered position of the lesioned tendon. It is precisely because of this positional adjacency that cell–cell cross-talks are more likely to occur among EC1, Mac, and diseased tenocytes. It has been reported that the tendon microenvironment of TSPC niches, including but not limited to ECM morphology, biochemical compositions, and stromal cells, is crucial for TSPC proliferation and differentiation [43]. Overall, EC1 and Mac changed the TSPC niche and make the microenvironment in the tendon more favorable for the differentiation of TSPC into chondrocytes and osteocytes. Thus, we agree with the concept that the variation of the tendon microenvironment is an important cause of tendinopathy. In the future, we may pay attention to methods of elimination of Mac and EC1 in the diseased microenvironment in our research.

One limitation of our research was the sample representation. During the design of this study, we only partitioned samples into two health states: normal and diseased. If we distinguish the disease severity of the tendon specimens, we could establish more fine-grained links between the molecular profiles and the disease. Our research focuses on the pathogenesis of tendinopathy, so we strive to analyze the differences between diseased and normal tissues and seek molecular explanations for the occurrence and development of tendinopathy at the single-cell level. In contrast, we have studied fewer transcriptional events in normal samples, so some information associated with normal tendon development may be missed.

## Conclusions

In summary, we systematically profiled the cellular heterogeneity in the normal and lesioned tendons and reported the molecular pathological mechanisms of tendon injury and microenvironmental alterations during tendinopathy progression using scRNA-seq and spatial transcriptome sequencing. We demonstrated at the single-cell level that tendon injury is accompanied by the formation of tendon HO. From the study, we believe that blocking the CD44 receptor on diseased tenocytes or knocking down the SPP1 gene in macrophages in the pathological environment may delay the occurrence and development of tendon HO, thus achieving a therapeutic effect on tendinopathy. We should give more attention to the abnormally increased endothelial cells and macrophages in damaged tendons, which alter the tendon microenvironment and are responsible for tendon HO. Future research should focus on methods to targeted eliminate these two disease-causing cells. These results will enable other investigators to identify additional tendinopathy-associated changes in a cell type-specific manner in future study. This will likely facilitate mechanistic and functional investigations into the role of specific genes in relevant microenvironmental homeostasis-regulated cell types in tendinopathy.

## Methods

### Human tendon cell sample preparation

The tendon specimens were collected from four patients with rotator cuff injury (diseased tendons) and four patients with trauma (normal tendons). This study was reviewed and approved by our University Ethics Committee (Ethics Committee on Biomedical Research, West China Hospital of Sichuan University No. 2020–921), and all procedures complied with the Helsinki Declaration. Participants gave informed consent to participate in the study.

All specimens removed were placed immediately in Iscove’s modified Dulbecco’s medium (IMDM) free of antibiotics and FBS under 4 °C. The tendons were rinsed in 1 × PBS and then were cut into 1-mm^3^ pieces. Collagenase type I (SCR103, Sigma-Aldrich) was used to digest the tissues under 37 °C for 60 min. After that, Ham’s F-12 media containing 10% FBS was added to stop the process of digestion and then the digested tissue passed through a 100-µm cell strainer. The dissociated cells were washed and resuspended for 10 × Genomics Chromium™ (Single Cell 3’library and Gel Bead Kit v2). According to the manufacturer’s instructions, single-cell suspension was subjected onto a 10 × Genomics Chromium Controller machine for gel beads-in-emulsion (GEM) construction. After forming normal GEMs, GEMs were oil-treated and within each oil droplet, mRNA were released from lysed cells, reverse transcribed to cDNA, barcode labeled, and sequenced. The final single-cell transcriptome library pool was reflected to the human reference genome GRCh38 and quantified by CellRanger 6.1.2.

### Immunohistochemistry

Immunohistochemical staining was performed as previously described [44]. Tissue slices were deparaffinized and rehydrated. After that, microwave was used to retrieve the antigen. Next, we used 3% hydrogen peroxide to block the endogenous peroxidase activity and 5% BSA to block the non-specific binding sites under room temperature. The primary antibodies were incubated with slices overnight at 4 °C. The next day, an appropriate HRP-conjugated secondary antibody was used to incubate with sections and counterstained with hematoxylin. Immunohistochemistry was done using the following primary antibodies: NR2F2 (A10251; Abclonal), EGR1 (55,117–1-AP; Proteintech), SOX5 (A6985; Abclonal), COMP (A13963; Abclonal), Collagen X (ab49945; Abcam), POSTN (A14556; Abclonal), CD68(A13286; Abclonal), and CD31(ab9498; Abcam).

### Multiplex immunofluorescence (OPAL™) staining

Tissue slices were deparaffinized and rehydrated and then heat-induced antigen retrieval was used. After that, various cell marker primary antibodies were incubated with the paraffin slide of tendon tissue to conduct the continuous staining with the Opal Polaris Multiple-Color Manual IHC Kit (NEL861001KT). We used different primary antibodies to simultaneously label macrophage (CD68), endothelial cell (CD31) and diseased tenocyte (POSTN) on the same tissue slide. DAPI was used to stain cell nuclei. We used automated staining system (BOND-RX, Leica Microsystems, Vista, CA) to perform the chromogen-based multiplex immunohistochemistry labeling. CD31 (ab9498) was purchased from Abcam, and POSTN (A14556) and CD68 (A13286) were purchased from ABclonal. The Opal Polaris dyes were used to pair with these antibodies containing fluorophores for tyramide signal amplification (TSA) to enhance sensitivity. The sequence of labeling for detecting each marker was optimized: POSTN (Opal 570), CD68 (530), CD31 (Opal 690), and DAPI.

### Single-cell RNA-seq data processing

CellRanger (version 6.1.2) coupled with human reference genome version GRCh38 was used with default settings to process FASTQ files acquired from 10 × Genomics for each sample. Meanwhile, to avoid the effects of doublets, Doublefinder was employed to remove doublets from each sample, following a doublet rate of 0.075. The output-filtered gene expression matrices were read using the *Read10X* function from the Seurat package (Version 4.0.4). The *Merge* function was utilized to merge all sample data into a collective object, and the *RenameCells* function was used to ensure that the cell name is unique. We then conducted comprehensive quality control steps. Briefly, low-quality cells were filtered if they satisfied the following requirements: (1) > 4000 or < 200 genes and (2) > 10% mitochondrial content. After filtering, we obtained 34,736 cells for subsequent analysis. We employed the “LogNormalize” method to normalize the gene expression levels and setting a scale factor of 10,000. The top 2000 most variable genes were defined as highly variable genes (HVG) for follow-up work using the *FindVariableFeatures* function. In order to eliminate unwanted sources of variation, the *ScaleData* function was performed to regress out number of genes, unique molecular identifiers (UMI), and percent mitochondrial content. We reduced the dimensionality of this dataset by using the principal component analysis (PCA) on HVG, and the first 30 principal components were identified. *RunFastMNN* function in the SeuratWrappers package (version 0.3.0) was employed to remove the batch effect between the samples. We performed the clustering analysis according to the edge weights between any two cells and a shared nearest-neighbor graph generated from the Louvain algorithm, which were respectively embedded in the *FindNeighbors* function and the *FindClusters* function. The clusters were visualized by using t-distributed stochastic neighbor (t-SNE) embedding. To identify the highly expressed markers of each cluster and differentially expressed genes, we applied the *FindAllMarkers* function with default non-parametric Wilcoxon rank sum test with Bonferroni correction.

### Cell–cell interaction analysis

We employed CellphonedDB to explore the cell–cell communication and ligand-receptor pairs between all major cell types. Circlize package (version 0.4.14) and *Heatmap_plot* function from CellphoneDB were utilized to display the frequency of interactions between two cell subpopulations. Potential interaction strength between two cell subsets, which was predicted based on the average expression of ligand-receptor pairs was mapped by using the *dot_plot* function and pheatmap package (version 1.0.12).

### NicheNet analysis

We also used NicheNet (Version 1.0.0) to identify potential ligands of EC1 and Mac, which drive the phenotype of diseased TCs. Compared with normal TCs, the top 100 highly expression genes ordered by log2FC in diseased TCs were regarded as potential targets. Mac and EC1 are sender cells, while diseased TCs are treated as target cells. Genes with a positive rate of more than 10% expressed in EC1, Mac, and diseased TCs were deemed as potential ligands or target genes. The *active_ligand_target_links* function was employed to examine the potential intensity of regulation between ligand and target.

### iTALK analysis

We also used the iTALK package to reveal the intercellular regulation at the cytokine and chemokine levels in the normal and disease groups, respectively. We inputted the raw count matrix of macrophage, DC, T cells, and TSPC and also included the normal (TC1–6) and diseased tenocytes (TC7–10). Only the first 100 regulatory effects were selected and displayed. If the ligand was derived from an immune cell and the receptor was from TSPC or TC cell, we marked this regulatory effect with a red line, and the others with a black line.

### Pseudotime trajectory analysis

To explore the potential dynamic processes of tenocyte functional changes during progression from normal to diseased cells and determine the potential lineage differentiation in TSPC and TC1–10, we applied the Monocle (version 2.22.0) algorithm. The gene-cell matrix of UMI counts was treated as the input to Monocle, and then, we used the NewCellDataSet function to create a new object for monocle with a transcript count data of the included cell populations. After that, we applied the *differentialGeneTest* function to determine the remarkably different genes over time. The *differentialGeneTest* function and “fullModelFormulaStr” option “ ~ sm.ns(Pseudotime)” were used to identify pseudotime-dependent genes, and smooth expression curves were derived from the *plot_pseudotime_heatmap* function. Ggridges package (version 0.5.3) was employed to compute the frequency of the cell distribution in different groups along the trajectory.

### Enrichment analysis

We performed GO and KEGG enrichment analysis based on genes associated with pseudotime trajectory or differentially expressed in the disease and control groups using the clusterProfiler package (version 3.0.4) with default settings. The EnrichIt function from the escape R package (version 1.4.0) was used to perform the enrichment analysis for EC1–5 and immune clusters with 50 hallmark gene sets in the MSigDB databases. The non-parametric and unsupervised algorithm named gene set variation analysis (GSVA) package (version 1.14.1) was used to assess the M1 and M2 scoring of Mac in normal and diseased groups and Dis-TC scoring, Mac scoring, and EC1 scoring in our spRNA-seq dataset.

### SCENIC analysis

All tenocyte subclusters and TSPC were further typed into the Single-Cell rEgulatory Network Inference and Clustering analysis (SCENIC) package (version 1.2.4) and then sorted based on clusters. The *geneFiltering* function with default settings was applied to remove genes with low expression levels or low positive rates, and only the genes matched the Rcis Target databases would be retained for subsequent analysis. GENIE3 was used to examine the relationship of transcription factor to the potential target. We selected 24,453 motifs from the cisTarget Human motif database v9 for enrichment of gene signatures, which pruned for targets according to cis-regulatory cues with default settings. The enrichment of regulons across incorporated individual cells was identified by the “aucell” positional argument, and we applied the pheatmap package (version 1.0.12) to visualize the result.

### Gene regulatory network analysis

To reveal the functional association, we constructed a gene regulatory network for the normal tenocytes (TC1–6) and the diseased tenocytes (TC7–10). We firstly eliminated the genes expressed in fewer than 25% of cells and the value of logFC < 0.25 and then selected the genes whose *q* value < 0.01, leaving us with 1049 genes for the network analysis. The vegdist function from the vegan package (version 2.6–5) was used to calculate the Jaccard index of 1049 genes in tenocytes with the jac method, and we next filtered out genes whose Jaccard index < 0.7. The network was then visualized using the force-directed layout algorithm in the open-source platform Cytoscape (version 3.8.0).

### Spatial transcriptome sequencing data processing

The bcl2fastq method was applied to transform raw base call (BCL) files into FASTQ reads. Reads were aligned to the human reference genome GRCh38-2020 using the Space Ranger (version 1.3.1) software, and then with the help of Read10X_h5 and CreateSeuratObject function, we created an object with the output of Space Ranger. *Read10X_Image* function was used to load the H&E image data, and the *SCTransform* function was used to normalize the dataset. The expression level of a single gene at a spatial location was visualized by using the *spatialFeaturePlot* function. To localize the macrophages, EC1, and normal and diseased TC cells of our interest in the single-cell data to tissue samples of the spatial transcriptome, we first calculated marker genes separately for the incorporated cell populations. For macrophage and EC1, we treated the top 50 highly expressed genes, which were identified with the *FindAllMarkers* function from the Seurat package, as the signatures, respectively. For normal and diseased TC cells, we also selected the top 50 differently expressed genes between controls and patients, respectively. We also applied the GSVA package (Version 1.14.1) to assess the above four signature expression scores in each well of the spatial transcriptome. To further integrate the single-cell and spatial transcriptome sequencing, the SPOTlight and NMF packages were used to infer the deconvolution scores of each cluster from single-cell reference for spatial transcriptomics capture locations (spots). Markers of each cluster in single-cell data were identified with the *FindAllMarkers* function, and the top 2000 highly variable genes were demonstrated by the *VariableFeatures* function with default settings. The *SPOTlight* function was performed for training the NMF model and deconvoluting mixture data. Finally, we could visualize the cell type proportions as sections of a pie chart for each spot through the *plotSpatialScatterpie* function.

### Statistical analysis

All the experiments were performed at least three times. Non-parametric Wilcoxon rank sum test was used to analyze the differences between the two groups. All statistical analyses were performed in R or GraphPad Prism (version 5.0). Statistical significance was defined as *p < 0.05, **p < 0.01, and ***p < 0.001.

## Supplementary Information

**Additional file 1:** **Table S1. **Donor and sample information.

**Additional file 2:** **Fig. S1. **Quality control process. A: Working and quality control pipeline in this work. The bar graph shows the cells that remained after each quality control step. Quality control was performed with doublets of each sample, number of genes per cell and percent of mitochondria gene. B and C: The box plot showing the distribution of detected total UMIs per celland gene numbersof the single cells in each of the 8 tendon samples.

**Additional file 3:** **Table S2.** Differentially expressed genes of each cluster.

**Additional file 4:** **Table S3.** Differentially expressed genes of different tenocyte subcluster.

**Additional file 5:** **Table S4. **Enrichment analysis of differentially expressed genes in normal and disease tenocytes.

**Additional file 6:** **Fig. S2. **Supplementary details. A and B: GSEA showing enrichment of pathways between tenocytes in diseased tissue and normal tissue. C: Heatmap showing the results of enrichment analysis of 50 hallmarker gene sets among T cell, DC and Mac in normal and diseased tissues. D and E: Cell distribution along normalor diseasedevolution trajectory. F: Analysis of cell interaction strength in spatial transcriptome of normaland degeneratedsamples. G: Immunohistochemical staining of POSTN, CD68 and CD31 in lesioned tendon.

**Additional file 7:** **Table S5. **Differentially expressed genes of different endothelial cell subcluster.

**Additional file 8:** **Fig. S3. **The control immunofluorescence images. A and B: The control immunofluorescence photosof normaland diseasedtendons.

## Data Availability

All data generated or analyzed during this study are included in this published article, its supplementary information files, and publicly available repositories. Data are available in a public, open-access repository. The single-cell RNA-seq data, spatial RNA-seq data, and cluster annotations are available at GSA for humans (https://ngdc.cncb.ac.cn/gsa-human/) with the accession number HRA002325.

## Abbreviations

scRNA-seq: Single-cell RNA sequencing
spRNA-seq: Spatial RNA sequencing
MRI: Magnetic resonance imaging
t-SNE: T-distributed stochastic neighbor embedding
TC: Tenocyte
DC: Dendritic cell
Mac: Macrophage
SMC: Smooth muscle cell
RBC: Red blood cell
TSPC: Tendon stem/progenitor cell
EC: Endothelial cell
DEGs: Differentially expressed genes
ECM: Extracellular matrix
SCENIC: Single-cell regulatory network inference and clustering
TFs: Transcription factors
GO: Gene Ontology
KEGG: Kyoto Encyclopedia of Genes and Genomes
GSEA: Gene set enrichment analysis
UMAP: Uniform manifold approximation and projection
GSVA: Gene set variation analysis
