# Supplementary figures and images for: Single-cell and spatial transcriptomics reveal changes in cell heterogeneity during progression of human tendinopathy
Source: BMC Biol. 2023 Jun 6;21:132. doi: 10.1186/s12915-023-01613-2 (PMC10246392; doi:10.1186/s12915-023-01613-2)

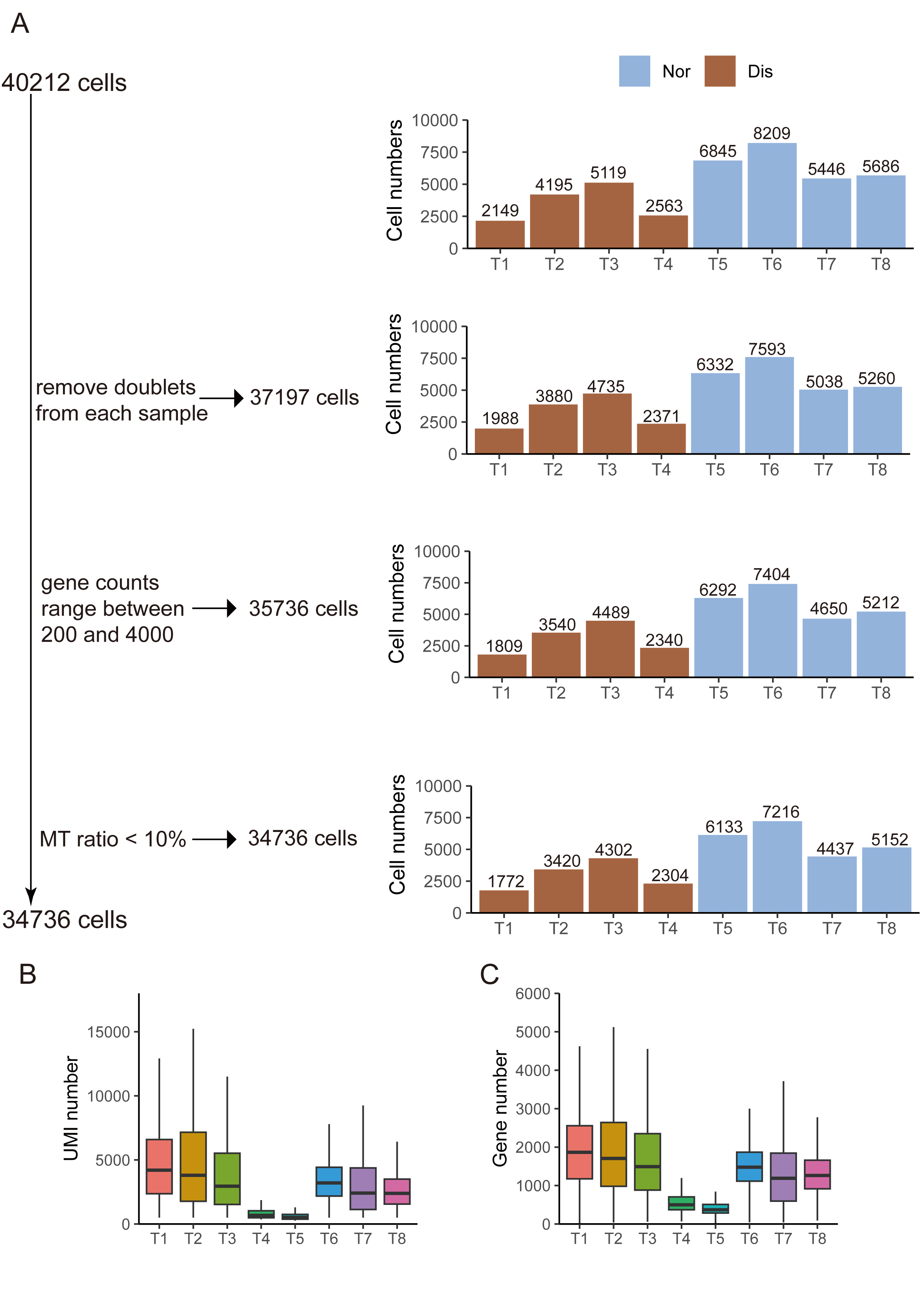

Supplement: Supplementary file 2 — Additional file 2: Fig. S1. Quality control process. A: Working and quality control pipeline in this work. The bar graph shows the cells that remained after each quality control step. Quality control was performed with doublets of each sample, number of genes per cell and percent of mitochondria gene. B and C: The box plot showing the distribution of detected total UMIs per celland gene numbersof the single cells in each of the 8 tendon samples. [file 12915_2023_1613_MOESM2_ESM.tif]

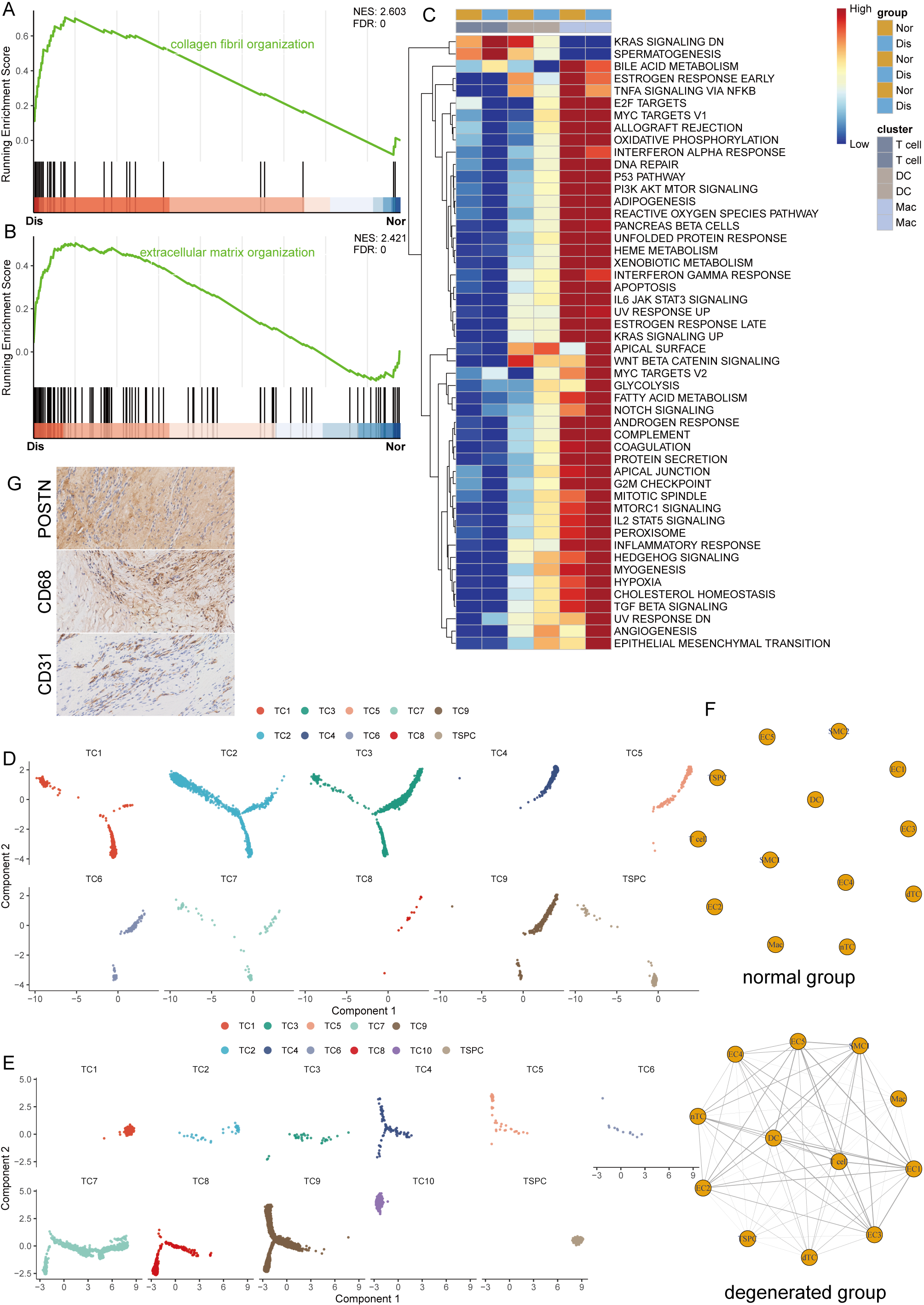

Supplement: Supplementary file 6 — Additional file 6: Fig. S2. Supplementary details. A and B: GSEA showing enrichment of pathways between tenocytes in diseased tissue and normal tissue. C: Heatmap showing the results of enrichment analysis of 50 hallmarker gene sets among T cell, DC and Mac in normal and diseased tissues. D and E: Cell distribution along normalor diseasedevolution trajectory. F: Analysis of cell interaction strength in spatial transcriptome of normaland degeneratedsamples. G: Immunohistochemical staining of POSTN, CD68 and CD31 in lesioned tendon. [file 12915_2023_1613_MOESM6_ESM.tif]

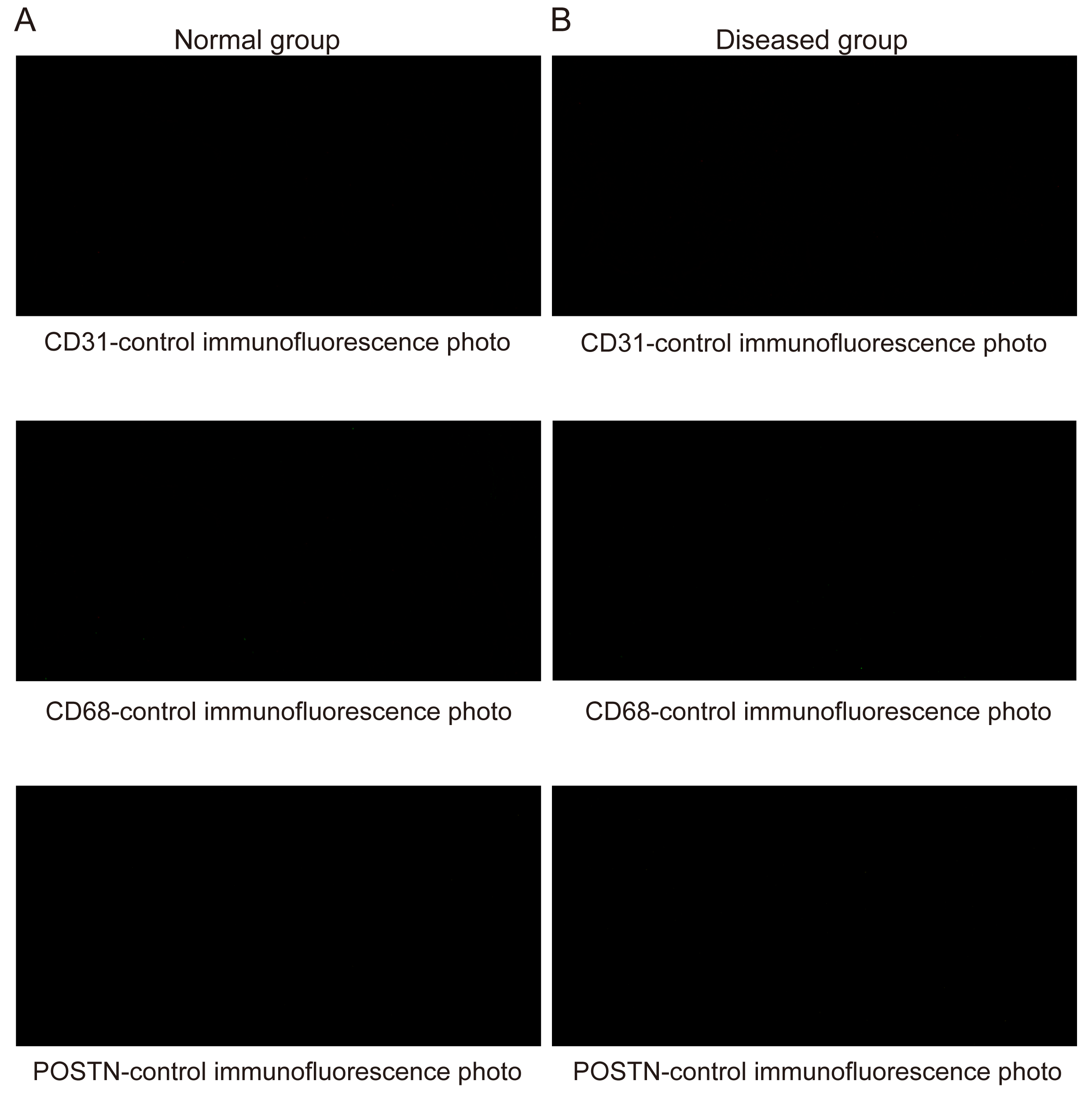

Supplement: Supplementary file 8 — Additional file 8: Fig. S3. The control immunofluorescence images. A and B: The control immunofluorescence photosof normaland diseasedtendons. [file 12915_2023_1613_MOESM8_ESM.tif]
